# A Multi‐Responsive Hydrogel Combined With Mild Heat Stimulation Promotes Diabetic Wound Healing by Regulating Inflammatory and Enhancing Angiogenesis

**DOI:** 10.1002/advs.202408783

**Published:** 2024-10-22

**Authors:** Fanjia Dai, Jiaying Zhang, Fengjiao Chen, Xianwu Chen, Celine Jessica Lee, Hongze Liang, Lingling Zhao, Hui Tan

**Affiliations:** ^1^ School of Material Science and Chemical Engineering Ningbo University Ningbo 315211 China; ^2^ Center for Child Care and Mental Health Shenzhen Children's Hospital Affiliated to Shantou University Medical College Shenzhen 518026 China; ^3^ The Affiliated Hospital of Medical School Ningbo University Ningbo 315211 China; ^4^ Queensland Children's Hospital QLD 4101 Australia

**Keywords:** angiogenesis, anti‐inflammatory, antioxidant, diabetic wound healing, hydrogel dressing, mild heat stimulation

## Abstract

The repair of diabetic wound still encounters huge challenges, such as disordered inflammatory regulation and impaired neovascularization. Here, a pH/ROS/glucose responsive and photothermal hydrogel is developed for diabetic wound healing. The hydrogel is formed through cross‐linkage between phenylboronic acid‐modified carboxymethyl chitosan (CMCS‐PBA) and oxide dextran (OXD), utilizing Schiff base and phenylboronate ester bonds. Additionally, insulin‐like growth factor 1 C domain (IGF‐1C) and deferoxamine‐loaded polydopamine nanoparticles (D@P) are incorporated into the hydrogel. The hydrogel demonstrates sustained drug release, excellent photo thermal effect, prominent antioxidant, antibacterial and anti‐inflammatory activities, desirable mechanical and tissue adhesive properties, enhanced tube formation, and cell migration. Furthermore, the hydrogel combined with mild heat treatment can regulate chronic inflammation by promoting the transformation of macrophages from M1 phenotype to M2 phenotype and enhance angiogenesis by up‐regulating the expression levels of angiogenesis‐related factors such as hypoxia‐inducible factor‐1 alpha (HIF‐1α), vascular endothelial growth factor (VEGF), CD31, and α‐SMA, thus greatly accelerates the wound healing in streptozotocin (STZ)‐induced diabetic mice. Therefore, this multi‐responsive and multifunctional hydrogel holds potential as a therapeutic strategy for diabetic wounds.

## Graphical abstract

A pH/ROS/glucose multi‐responsive composite hydrogel with good photothermal effect, and antioxidant, and anti‐inflammatory properties is designed for diabetic wound healing. The hydrogel combined with mild heat treatment can regulate chronic inflammation by promoting the M1/M2 macrophage transformation in vivo, promote angiogenesis by up‐regulating the expression levels of HIF‐1α, VEGF, CD31, and α‐SMA, and thus accelerate the diabetic wound healing.

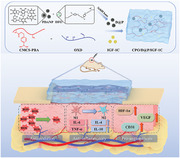

## 1 Introduction

The treatment of diabetic wounds poses significant challenges in clinical settings and profoundly impacts patients' quality of life.^[^ ^1^ ^]^ Wound healing can generally be divided into four stages including hemostasis, inflammation, proliferation, and tissue remodeling.^[^ ^2^ ^]^ Acute wounds usually progress through these stages in a regular and orchestrated way, eventually healing within a few weeks. However, this process is disrupted in diabetic wounds due to the harsh wound environment such as sustained hyperglycemia and persistent inflammation.^[^ ^3^ ^]^ The specific high‐glucose environment disrupts the physiological cascade response of wound healing and inhibits the transition from pro‐inflammatory M1 macrophage phenotype to anti‐inflammatory M2 macrophage phenotype, resulting in the prolonged release of proinflammatory factors and elevated levels of reactive oxygen species (ROS) and proteinases in the wound bed.^[^ ^4^ ^]^ Elevated ROS and proteinase levels lead to extracellular matrix (ECM) destruction and growth factor degradation, thereby attracting more inflammatory cells to the lesion.^[^ ^2^ ^]^ In addition, in a hyperglycemic environment, wounds are more likely to be infected by bacteria and other microorganisms, and even form biofilms.^[^ ^5^ ^]^ Persistent infection triggers the release of inflammatory chemokines and proteases, impairs immune cell function, and amplifies the inflammatory response, thereby perpetuating a detrimental cycle of “infection‐inflammation‐destruction” that hinders wound healing.^[^ ^2^ ^]^ Furthermore, the high blood glucose levels in diabetes also lead to the formation of advanced glycation end products (AGEs),^[^ ^6^ ^]^ which subsequently trigger inflammation and ROS production, resulting in impaired neovascularization.^[^ ^7^ ^]^ Blood vessels can supply oxygen and nutrients for cells in the wound sites, and maintain the growth of regenerative tissues. Therefore, compromised neovascularization can significantly impede wound healing function and further exacerbate wound deterioration.^[^ ^8^ ^]^ Thus, relevant management of the diabetic wound microenvironment, including regulation of blood glucose levels, modulation of the inflammatory response, alleviation of ROS damage, inhibition of bacterial infection, and promotion of angiogenesis, is imperative for effective diabetic wound healing.

Hydrogel dressings have garnered significant attention in the field of wound repair due to their ability to create a moist microenvironment, facilitate nutrient and metabolite exchange, promote cell migration, and ultimately aid in wound healing.^[^ ^9^ ^]^ Moreover, hydrogels can be utilized as carriers for various bioactive substances such as growth factors, drugs, cells, and bioactive materials. These substances can play an intervention role in anti‐inflammatory processes, antioxidant activities, blood glucose control, and angiogenesis to further enhance the healing of diabetic wounds. Previous studies have demonstrated that modulating blood glucose levels effectively promotes wound healing in diabetic mice. In our previous work, a pH and glucose dual‐responsive injectable hydrogel encapsulating insulin and fibroblasts was developed through the cross‐linking of Schiff base and phenylboronate ester for diabetic wound healing.^[^ ^10^ ^]^ Results indicated that the hydrogel accelerated the healing process of diabetic wounds by reducing the blood glucose levels due to the sustained release of insulin. However, glycemic control alone is insufficient due to the complex physiological microenvironment present in diabetic wounds. Considering that the prolonged inflammatory phase in diabetic wounds seriously hampered the processing of wound healing into the proliferation stage, several anti‐inflammation strategies have been developed including scavenging excessive ROS via antioxidants and promoting M2 macrophage polarization. Liu and coworkers designed a hemostatic and anti‐inflammatory dual­cross­linked hydrogel consisting of methacryloyl‐substituted Bletilla Striata polysaccharide and gelatin methacrylate.^[^ ^1^ ^]^ The hydrogel can promote diabetic wound healing by modulating the wound inflammation microenvironment due to the functionality of Bletilla Striata polysaccharide for macrophage phenotypes regulation. Furthermore, guided angiogenesis is also important for diabetic wound healing, because neovascularization was seriously impaired due to the elevated inflammation and ROS. Yuan and coworkers developed an M2 macrophage‐polarized anti‐inflammatory hydrogel to promote diabetic wound healing mainly by enhancing angiogenesis and regulating the macrophage phenotype.^[^ ^11^ ^]^ The integration of deferoxamine (DFO)‐loaded mesoporous polydopamine nanoparticles into the hydrogel imparts photothermal properties to facilitate proangiogenic efficacy. The use of a natural active compound of epigallocatechin‐gallate and high‐molecular‐weight hyaluronic acid endows the hydrogel with good anti‐inflammatory and antibacterial efficacy to facilitate diabetic wound healing. Therefore, the development of multifunctional hydrogels is crucial for diabetic wounds, which can promote diabetic wound healing through multi‐dimensional synergistic effects encompassing antioxidation and antibacterial effects, regulation of inflammatory response, and promotion of angiogenesis.

**Scheme 1:**
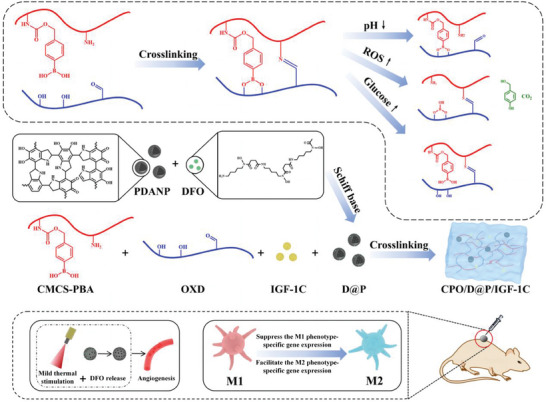
Synthesis diagram of CPO/D@P/IGF‐1C hydrogel and mechanism of diabetic wound healing promoted by the hydrogel combined with mild heat stimulation.

In this work, we proposed to design a multifunctional hydrogel, that can regulate the blood glucose levels in diabetes, prevent bacterial invasion, modulate the inflammation response of diabetic wounds to impel wound progression, enhance angiogenesis to provide nutrients and oxygen in the wound, and thus synergistically promote the repair of diabetic wound. Hence, a pH/ROS/glucose multi‐responsive hydrogel with antioxidant, anti‐inflammatory, and photothermal properties was developed for diabetic wound healing. The multifunctional hydrogel was prepared through the imine and phenylboronate ester crosslinking of CMCS‐PBA and OXD, and IGF‐1C and DFO‐loaded polydopamine nanoparticles were incorporated into the hydrogel. IGF‐1C is the C domain peptide of IGF‐1 with a 12‐amino acid sequence GYGSSSRRAPQT, which has been identified as the active region of IGF‐1 protein. Studies have demonstrated that IGF‐1C can regulate glucose metabolism,^[^ ^12^, ^13^ ^]^ inhibit oxidative stress, and be relevant to the promotion of wound healing.^[^ ^14^, ^15^ ^]^ DFO is an FDA‐approved iron chelator in the clinic, which is proved to promote vascularization by preventing the degradation of HIF‐1α and upregulating the expression of VEGF.^[^ ^16^ ^]^ DFO can also reduce the excess free radicals in the wounds, thereby preventing wound tissue damage and controlling the inflammation response. Polydopamine nanoparticles (PDANP) were used as biocompatible carriers for DFO to control the release behavior. DFO can be loaded in the PDANP through surface adsorption as well as the formation of imine bonds between the abundant quinone group in PDANP and the amino group in DFO. In addition, PDANP is an excellent photothermal material with significant photothermal capacity, which can effectively convert near‐infrared lasers into heat.^[^ ^17^ ^]^ Recent studies have confirmed that a mild heat treatment (40 and 41 °C) can promote the transformation of M1 macrophage to M2 macrophage and enhance neovascularization.^[^ ^18^, ^19^ ^]^ What's more, the antibacterial capability of hydrogels derived from the inherent antibacterial activity of CMCS can be further enhanced through the combination of hydrogel and photothermal effect. As a result, this multifunctional hydrogel will break the vicious cycle of “infection‐inflammation‐destruction” through a multi‐dimensional synergistic action, including scavenging ROS to reduce oxidative stress, preventing bacterial invasion to inhibit infection, and promoting the polarization of M2 macrophage to regulate the expression levels of inflammatory factors, so as to regulate the chronic inflammatory microenvironment of diabetic wounds. After administration of the hydrogel dressings, in the environment of the weak acid, high blood glucose, and elevated ROS levels on diabetic wounds, the dissociation of imine and phenylboronate ester leads to the accelerated release of IGF‐1C and DFO from the hydrogel, thus to accelerate the diabetic wound healing by reducing the blood glucose, regulating inflammatory, and promoting angiogenesis (**Scheme** **1**).

## 2 Results and Discussion

### 2.1 Synthesis and Characterization of CMCS‐PBA and OXD

**Figure 1:**
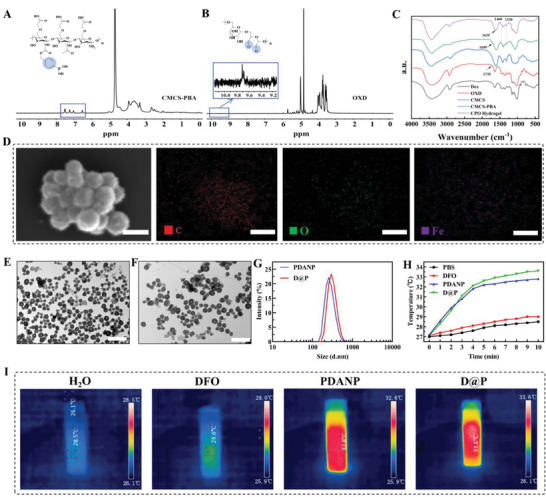
Characterization of CMCS‐PBA, OXD, PDANP, and D@P. ^1^H NMR spectra of CMCS‐PBA (A) and OXD (B); (C) FTIR spectra of CMCS, CMCS‐PBA, dextran, OXD, and CPO hydrogel; (D) SEM image and EDS element mappings of the C, O and Fe signals in Fe^3+^ coordinated D@P; TEM images of PDANP (**E**) and D@P (F); (G) The size distribution of PDANP and D@P; (H) Temperature elevation curves of PBS, DFO, PDANP and D@P under 808 nm laser irradiation; (I) Infrared thermographic images of PBS, DFO, PDANP and D@P under 808 nm laser irradiation at 10 min. Scale bars: 200 nm in (D), 1000 nm in (E) and (F).

*p*‐hydroxymethyl phenyboric acid group was introduced into the CMCS skeleton as a ROS and glucose‐responsive moiety to form CMCS‐PBA. CMCS‐PBA was synthesized by the aminolysis of NBC with the amino group on CMCS and the subsequent hydrolysis of borate ester. As shown in the ^1^H NMR spectra of CMCS‐PBA (**Figure** **1**A), the peaks that appeared between 6.6 and 7.7 ppm were assigned to the protons of the benzene ring in phenylboronic acid.^[^ ^20^ ^]^ In the FTIR spectra of CMCS‐PBA (Figure 1C), the weak peak at 1330 and 1460 cm^−1^ assigned to the stretching vibration of B‐C, and the peak at 1690 cm^−1^ assigned to the stretching vibration of C═O in amide was increased due to the formation of CSPBA. All the results indicated that phenylboronic acid has been successfully conjugated to CMCS.

OXD bearing aldehyde group was synthesized by oxidizing the vicinal hydroxyl groups of dextran, which can serve as a macromolecular cross‐linker for polysaccharides containing free amino groups to form the hydrogel. The ^1^H NMR spectra of OXD are shown in Figure 1B. The characteristic proton peak of the aldehyde group was observed at 9.7 ppm, and the peak is with low intensity due to the formation of hemiacetals, which is confirmed by the appearance of several peaks between 5.8 and 4.2 ppm, assigned to protons from different hemiacteal structures.^[^ ^21^ ^]^ In the FTIR spectra of OXD (Figure 1C), the peak at ≈1735 cm^−1^ assigned to the stretching vibration of C═O in the aldehyde group was also observed, suggesting the successful synthesis of OXD.

### 2.2 Synthesis and Characterization of PDANP and D@P

PDANP was prepared through a one‐pot method,^[^ ^22^ ^]^ and DFO was loaded into PDANP via Schiff base and surface adsorption to form D@P. The loading of DFO in D@P was further confirmed by coordinating D@P with Fe^3+^. As shown in the EDS elemental mapping images of Fe^3+^ coordinated D@P (Figure 1D), Fe was uniformly distributed in PDANP, indicating that DFO was successfully loaded in PDANP. PDANP and D@P had a hollow spherical structure as shown in the TEM images (Figure 1E,F). The average particle size of PDANP was 255 ± 8 nm, and the particle size of D@P was increased to 295 ± 11 nm after DFO loading (Figure 1G). The DFO loading efficiency was 34.1% measured by HPLC. The photothermal property of PDANP and D@P was tested. After 10 min of laser irradiation under 808 nm (1 W, 15 cm), the temperature of PBS, DFO, PDANP and D@P rose to 28.5, 29.0, 32.8 and 33.6 °C, respectively, as shown in Figure 1H,I. The results showed that PDANP had a good photothermal effect and the photothermal effect was not affected after DFO loading.

### 2.3 Preparation and Characterization of the Hydrogels

**Figure 2:**
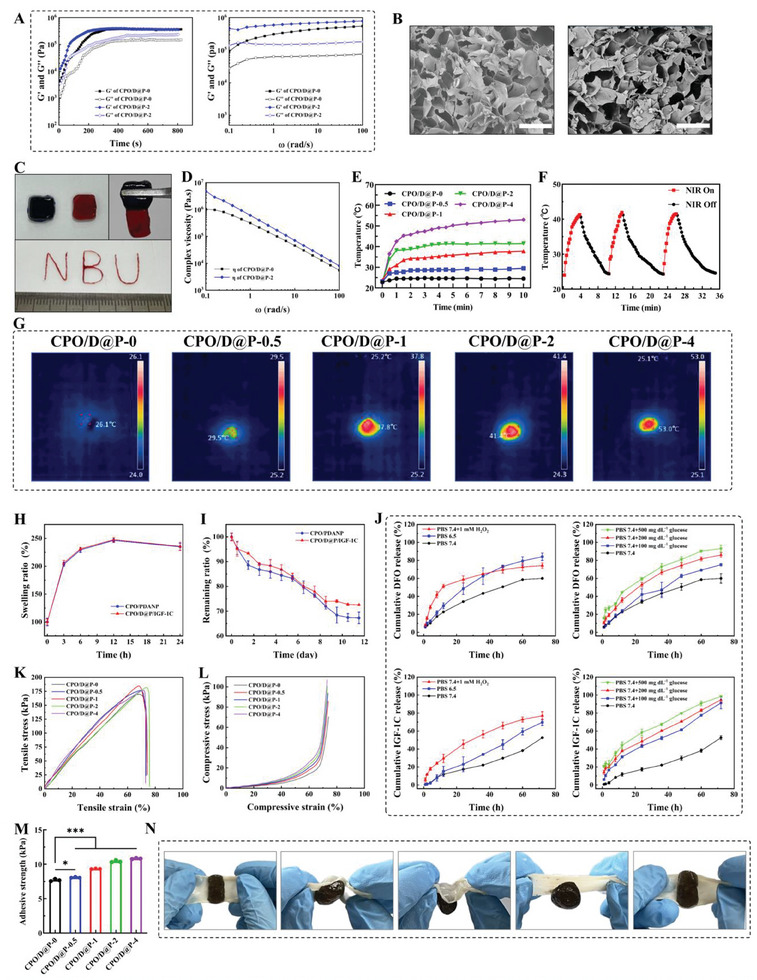
Rheology, morphology, self‐healing, injectable, photothermal, swelling and degradation properties, in vitro drug release behavior, mechanical and adhesive properties of the hydrogels. A) Time and frequency dependence of G' and G” of hydrogels; (B) SEM image of the section of freeze‐dried CPO (left) and CPO/D@P/IGF‐1C hydrogel (right), scale bar is 500 µm.; C) Self‐healing and injectable properties of hydrogels; D) Shear thinning properties of the hydrogels; E) Temperature elevation curves of CPO/D@P hydrogels with different D@P content under laser irradiation; F) Heating curve of CPO/D@P‐2 hydrogel for three laser on/off cycles. G) Infrared thermal images of CPO/D@P hydrogels containing different D@P content under 808 nm laser irradiation at 10 min. The swelling H) and degradation I) properties of hydrogels in PBS at 37 °C, n = 3; J) Cumulative release of DFO and IGF‐1C from the hydrogel at different pH, H_2_O_2_ and glucose concentrations, n = 4; K) Tensile stress‐strain profile of the hydrogels; L) Compressive stress‐strain profile of the hydrogels; M) Adhesive strengths of the hydrogels, Tukey's multiple comparisons test, n = 3, ^*^ *p* < 0.05, ^***^ *p* < 0.001; (N) Photographs of the CPO/D@P‐2 hydrogel adhered to the skin of mice.

CPO hydrogel was formed through the cross‐linkage of imine and phenyl borate (Scheme 1). Imine bonds were formed by the reaction between amino groups of CMCS‐PBA and aldehyde groups of OXD, and phenyl borates were formed by the interaction between the phenylboronic acid group of CMCS‐PBA and the vicinal diol structure of OXD. As shown in the FTIR spectra of freezing dried hydrogel (Figure 1C), the weak peak at 1330 cm^−1^ assigned to the asymmetric extension of B‐O‐C proved the formation of a phenyl borate structure.^[^ ^23^, ^24^ ^]^ The peak at 1735 cm^−1^ assigned to the C═O stretch vibration of aldehyde groups disappeared and the peak at 1635 cm^−1^ appeared due to the formation of imine bonds.^[^ ^25^, ^26^ ^]^ The hydrogel formation was also confirmed by rheological analysis. As shown in **Figure** **2**A, G’ surpassed G’’ for the mixture of CMCS‐PBA and OXD solution, indicating a rapid gelation of the system. Both G’ and G’’ increased with time and reached a plateau within several minutes. CPO hydrogel consisting of 2 wt.% CMCS‐PBA and 2 wt.% OXD had a plateau G’ of ≈360 kPa, indicating good elasticity of the hydrogel. The addition of D@P had little influence on G’ of hydrogel at identical polymer concentrations, and CPO/D@P hydrogel consisting of 2 wt.% CMCS‐PBA, 2 wt.% OXD and 0.2 wt.% D@P had a similar plateau G’ (≈360 kPa). G’ was independent with the shear frequency once the hydrogel was formed stably, indicating good robustness of the hydrogel. SEM images of CPO and CPO/D@P/IGF‐1C hydrogel showed that the hydrogel network has a continuous porous microstructure (Figure 2B), which was conducive to the transfer and exchange of substances. The addition of D@P and IGCF‐1C showed little influence on the hydrogel network.

### 2.4 Self‐Healing and Injectable Properties of the Hydrogels

CPO hydrogels showed good self‐healing properties due to the dynamic cross‐linkage of imine and phenyl borate. As shown in Figure 2C, two pieces of CPO hydrogel merged into one hydrogel when they were placed next to each other for 5 min. When hydrogels are damaged by activities or external forces, the self‐healing properties of the hydrogels help to maintain their integrity. The viscosity of the CPO and CPO/D@P hydrogels decreased with increasing shear rate (Figure 2D), and this shear thinning property led to good injectability of the hydrogels. As shown in Figure 2C, the CPO hydrogel can be extruded from the syringe to write the word “NBU” on the cardboard. The injectable property of the hydrogel helps to fill irregular wounds and avoid unnecessary wound expansion.

### 2.5 Photothermal Performance of the Hydrogels

The photothermal effect of CPO/D@P hydrogels was evaluated under 808 nm NIR irradiation. The temperature of CPO/D@P hydrogels containing different amounts of D@P (0, 0.5, 1.0, 2.0 and 4.0 mg mL^−1^) exposed to laser irradiation (1 W, 15 cm, 10 min) reached 26.1, 29.5, 37.8, 41.4 and 53.0 °C, respectively (Figure 2E,G). The photothermal conversion efficiency of the CPO/D@P‐2 hydrogel was ascertained as 22.17% (Figure S1, Supporting Information), the value was similar to most reported photothermal materials,^[^ ^17^, ^22^, ^27^ ^]^ demonstrating the good photothermal effect of the CPO/D@P hydrogel. The photothermal stability of the CPO/D@P‐2 hydrogel was assessed by exposing the hydrogel to an alternated switching laser irradiation. As shown in Figure 2F, in the three cycles of NIR on and off tests, the temperature of CPO/D@P‐2 hydrogel reached 41.4 °C within several minutes when the NIR was on, and then cooled down to 25 °C when the NIR was off, indicating good photothermal stability of the CPO/D@P‐2 hydrogel. Studies showed that a mild heat (40 and 41 °C) could apparently promote the tube formation of endothelial cells and angiogenesis in hindlimb ischemic mice.^[^ ^11^ ^]^ Therefore, CPO/D@P‐2 hydrogel containing 2.0 mg mL^−1^ was used in the following experiments.

### 2.6 Swelling and Degradation Properties of the Hydrogels

The swelling and degradation properties of the hydrogel directly affect the exchange of nutrients, gases and metabolites. As shown in Figure 2H, the swelling ratio of CPO/PDANP hydrogels increased rapidly in the first 3 h (203%) and slowly reached equilibrium in 12 h (246%). CPO/D@P/IGF‐1C hydrogels demonstrated a quite similar swelling performance in a simulated physiological environment at 37 °C in PBS. The swelling ratio of CPO/D@P/IGF‐1C hydrogels increased rapidly in the first 3 h (206%) and slowly reached equilibrium in 12 h (248%). After the hydrogels reached the swelling equilibrium of expansion, the degradation performance of the hydrogels was evaluated. As shown in Figure 2I, a relatively rapid degradation rate was observed in the first 36 h, and the degradation rate was slowed in the later stages. The remaining percentage of CPO/PDANP hydrogels was 88.6 ± 1.7% at 48 h, and then gradually decreased to 67.3 ± 3.2% on day 12. For CPO/D@P/IGF‐1C hydrogels, the remaining percentage was 93.3 ± 1.3% at 48 h, and then gradually decreased to 72.5 ± 1.1% on day 12.

### 2.7 Drug Release Behavior of the Hydrogels

The in vitro drug release of IGF‐1C and DFO in CPO/D@P/IGF‐1C hydrogels was evaluated in PBS with different pH values, H_2_O_2_ and glucose concentrations. As shown in Figure 2J, sustained release of IGF‐1C and DFO in the hydrogels can be observed, and the drug release was accelerated at low pH, high H_2_O_2_ and glucose concentrations. At physiological conditions (pH 7.4), both IGF‐1C and DFO were released sustainedly from the hydrogels without obvious burst release, and the cumulative release was 60.0% and 52.6% at 72 h for DFO and IGF‐1C, respectively. The release of DFO in CPO/D@P/IGF‐1C hydrogels was faster at acidic pH, and high H_2_O_2_ and glucose concentrations. The release of DFO in CPO/D@P/IGF‐1C hydrogels was increased to 84.1% (pH 6.5), 75.2% (pH 7.4+100  g dL^−1^ glucose), 86.1% (pH 7.4+200  g dL^−1^ glucose), 93.2% (pH 7.4+500  g dL^−1^ glucose), and 74.3% (pH 7.4+1 mM H_2_O_2_) at 72 h. This is because that imine bond was unstable under acidic pH, and phenylboronate ester will preferentially combine with glucose in the presence of glucose^[^ ^28^ ^]^ and can also be oxidized by H_2_O_2_ and removed from the hydrogel network,^[^ ^29^ ^]^ which resulted in a much looser or even damaged hydrogel network. This means that the release of DFO was mainly caused by the cleavage of imine bonds and a much looser hydrogel network due to the dissociation of cross‐linkage in the hydrogels. Similarly, under conditions of acidic pH, high H_2_O_2_ and glucose levels, the release of IGF‐1C in CPO/D@P/IGF‐1C hydrogels was increased to 69.8% (pH 6.5), 91.4% (pH 7.4+100  g dL^−1^ glucose), 94.8% (pH 7.4+200  g dL^−1^ glucose), 98.6% (pH 7.4+500  g dL^−1^ glucose), and 77.0% (pH 7.4+1 mM H_2_O_2_) at 72 h, probably due to the much looser polymer network of the hydrogel resulted by the dissociation of imine and phenylboronate ester cross‐linkages. The lower pH, high H_2_O_2_ and glucose‐accelerated drug release indicated that the CPO/D@P/IGF‐1C hydrogels were suitable for diabetic wound treatment because of the acidic pH, and high H_2_O_2_ glucose levels in diabetic wounds microenvironment.

### 2.8 Mechanical and Tissue‐Adhesive Properties of the Hydrogels

Ideal mechanical properties and enhanced tissue adhesion are critical for hydrogel dressings, which can effectively avoid tearing, deformation and peeling of hydrogel dressings after being administrated to wounds. The tensile performance of the hydrogels is shown in Figure 2K. The stretchability of the hydrogels is 72.9–75.8%, which is very close to the ductility of the skin (60–75%).^[^ ^11^ ^]^ The compression performance of the hydrogels is shown in Figure 2L. The maximum compressive stress of CPO hydrogel is 70.5 kPa, and the maximum compressive stress of hydrogels increases with increasing PDANP content. The CPO/D@P‐2 hydrogel could withstand 73.3% compressive strain under a stress of 100.7 kPa. These results demonstrated that the hydrogels had satisfactory stretchability and compressibility.

Hydrogel dressings with desirable adhesion properties can adhere and integrate with the tissue to seal the wound area and prevent infection. Figure 2M showed that the adhesion strengths of CPO/D@P hydrogels were larger than that of CPO hydrogel (7.7 ± 0.2 kPa) due to the integration of PDANP, and the adhesion strengths of hydrogels increased with increasing PDANP content. The adhesion strengths of CPO/D@P hydrogels increased from 8.1 ± 0.1 to 10.8 ± 0.3 kPa as the PDANP content increased from 0.5% to 4%, which was larger than that of fibrin glue (Fibrin Glue, EVICEL Fibrin Sealant, ≈3 kPa).^[^ ^30^ ^]^ Additionally, CPO/D@P‐2 hydrogel showed excellent adhesion to the mice's skin surface without any external force. As shown in Figure 2N, the hydrogel adhered firmly to the freely twisted mice skin. The enhanced adhesion strength of CPO/D@P hydrogel was attributed to the adhesive force and cohesive force. The adhesive fore was due to the “tissue anchor” between amino and mercaptan groups on the tissue surface and quinone groups of PDANP in CPO/D@P hydrogel,^[^ ^31^ ^]^ and the improved cohesive force was attributed to the excellent mechanical property of the CPO/D@P hydrogel.^[^ ^30^ ^]^ These results indicated that the hydrogels had desired mechanical and adhesive properties, and can be applied in diabetic wounds.

### 2.9 Antioxidant Activity of the Hydrogels

**Figure 3:**
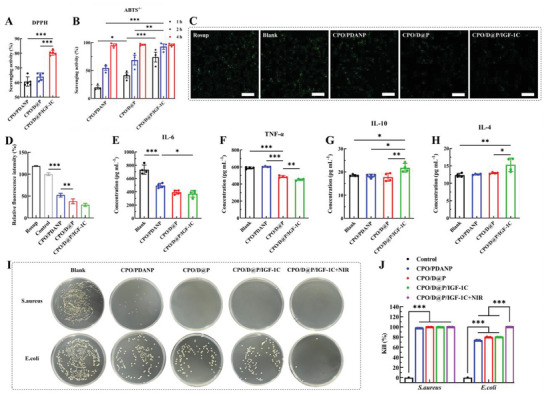
Hydrogels showed excellent antioxidant, anti‐inflammatory and antibacterical activities. DPPH (n = 5) A) and ABTS^+•^ (n = 3) B) radical scavenging efficiency of hydrogels; C) Images of ROS signals in HSF cells stimulated by H_2_O_2_ and subsequently treated with different hydrogel leaching solutions, scale bar is 500 µm; D) Quantitative analyze of the relative fluorescence intensity of DCFH‐DA (n = 3). The expression levels of IL‐6 E), TNF‐α F), IL‐10 G) and IL‐4 H) in the supernatant of LPS‐activated RAW264.7 macrophages treated with hydrogels, n = 4. I) Photographs of bacterial colonies after exposure to different hydrogels without or with NIR 808 nm. J) Killing ratio of bacteria, n = 5. Tukey's multiple comparisons test, ^*^ *p* < 0.05, ^**^ *p* < 0.01, ^***^ *p* < 0.001.

Excessive ROS accumulation has been identified in diabetic wounds and hampers wound healing by impairing the proliferative and remodeling phases of regeneration.^[^ ^3^ ^]^ Hydrogel dressings with antioxidant properties can effectively scavenge ROS, and alleviate oxidative damage, thus promoting diabetic wound repair. DPPH and ABTS^+•^ free radicals scavenging tests were used to evaluate the in vitro ROS scavenging ability of the hydrogels. As shown in **Figure** **3**A, the hydrogel system demonstrated good DPPH scavenging efficiency. After 4 h incubation, CPO/PDANP hydrogel showed obvious oxidation resistance with 60.7 ± 3.2% DPPH radical scavenging efficiency, and CPO/D@P hydrogel had a similar DPPH radical scavenging ability (63.9 ± 2.6%). Compared with CPO/PDANP and CPO/D@P hydrogels, the CPO/D@P/IGF‐1C hydrogel showed a much higher DPPH scavenging efficiency (80.3 ± 1.6%). In addition, the hydrogel system showed excellent ABTS^+•^ scavenging capability. As shown in Figure 3B, after 1 h of incubation, CPO/PDANP and CPO/D@P hydrogel have a scavenging ratio of ≈19.7 ± 5.1% and 41.3 ± 6.2%, respectively. CPO/D@P/IGF‐1C hydrogel showed a much higher ABTS^+•^ scavenging efficiency (73.9 ± 8.3%), indicating an outstanding radical scavenging capability of the hydrogel system. ABTS^+•^ scavenging efficiency of CPO/PDANP and CPO/D@P hydrogels increased with the incubation time. After 4 h incubation, the ABTS^+•^ scavenging efficiency was 95.3 ± 4.3% and 96.1 ± 1.9% for CPO/D@P and CPO/D@P/IGF‐1C hydrogel, respectively. The radical scavenging capability of the CPO/PDANP and CPO/D@P hydrogel was partly due to the D@P because the reductive groups of PDANP such as hydroquinone moieties, imine and catechol groups could remove free radicals,^[^ ^11^ ^]^ and partly due to the phenylboronate ester cross‐linkage of the hydrogel, which could consume ROS through the oxidization of phenylboronate structure.^[^ ^20^ ^]^ The antioxidant activity of the CPO/D@P/IGF‐1C hydrogel was attributed not only to the presence of PDANP but also to IGF‐1C. Because IGF‐1C could inhibit oxidative stress by decreasing superoxide and ROS levels.^[^ ^14^ ^]^

Furthermore, the ability of the hydrogel system to protect HSF cells from ROS damage was evaluated by the intracellular ROS scavenging test. HSF cells were stimulated with H_2_O_2_ (100 µM) to produce excessive ROS in cells, and the intracellular ROS was detected using the ROS indicator DCFH‐DA. As shown in Figure 3C,D, HSF cells treated with H_2_O_2_ exhibited visible fluorescence, indicating the production of excessive intracellular ROS. When incubated with CPO/PDANP and CPO/D@P hydrogel, HSF cells exhibited reduced green fluorescence relative to the H_2_O_2_ treatment group. Obvious fluorescence quenching was observed in HSF cells incubated with CPO/D@P/IGF‐1C hydrogels due to the excellent ROS scavenging ability of IGF‐1C. It is reported that IGF‐1 plays an important role in protection against age‐related oxidative damage.^[^ ^32^ ^]^ It can be proposed that the CPO/D@P/IGF‐1C hydrogel could attenuate the apoptotic cell death and senescence promoted by the oxidative stress and ROS accumulation in diabetic wound.

### 2.10 Anti‐Inflammatory and Antibacterial Activities of the Hydrogels

The high systemic blood glucose levels resulting from diabetes lead to up‐regulate expression of proinflammatory cytokines, which lead to elevated levels of proteases and destruction of ECM, and ECM destruction attracts more inflammatory cells, thus amplifying the inflammation cycle and results in prolonged inflammation.^[^ ^2^ ^]^ Thus, hydrogel dressing with anti‐inflammatory ability could promote diabetic wound healing. Therefore, the anti‐inflammatory ability of hydrogels was evaluated using LPS‐activated RAW264.7 macrophages and the expression levels of inflammatory cytokines were tested. As shown in Figure 3E–H, the CPO/D and CPO/D@P/IGF‐1C hydrogels exhibited good anti‐inflammatory activity, and the CPO/D@P/IGF‐1C hydrogel had the best in vitro anti‐inflammatory performance with a significantly reduced expression of IL‐6 and TNF‐α and a remarkable increased the expression of IL‐4 and IL‐10. These findings suggested that CPO/D@P/IGF‐1C hydrogel may be beneficial in addressing wound inflammation, as TNF‐α is a central pro‐inflammatory cytokine released early in the inflammatory response and drives the further involvement in the inflammatory signaling cascade (such as IL‐6),^[^ ^33^ ^]^ and IL‐6 is a key proinflammatory mediator that induces the release of proinflammatory cytokines and the chemotaxis of leukocytes in a wound.^[^ ^34^ ^]^ IL‐10 is a significant inflammatory cytokine that can suppress effector T cell responses and antagonize the effect of IL‐6,^[^ ^35^ ^]^ and IL‐4 is a typical anti‐inflammatory cytokine that can drive the differentiation of macrophages to M2 phenotypes and reduce inflammation.^[^ ^36^ ^]^ Studies showed that inflammatory reactions are associated with an initial increase in ROS levels, which further intensifies the inflammatory cascade activity.^[^ ^37^, ^38^ ^]^ Accordingly, the potent anti‐inflammatory ability of the CPO/D@P/IGF‐1C hydrogel was likely contributed to its robust antioxidant and ROS scavenging capacities. DFO has antioxidant capacity independent of iron chelation and can attenuate oxidative stress involved in inflammatory responses.^[^ ^39^ ^]^ IGF‐1C could promote the polarization of M2 macrophages and reduce inflammation.^[^ ^40^ ^]^

The special microenvironment of diabetic wounds increases the risk of bacterial infection, which may lead to prolonged inflammation and delayed wound healing. The antibacterial activities of CPO/PDANP, CPO/D@P, CPO/D@P/IGF‐1C and CPO/D@P/IGF‐1C+NIR were evaluated against *Escherichia coli* (*E. coli*, Gram‐negative bacterium) and *Staphylococcus aureus* (*S. aureus*, Gram‐positive bacterium) in vitro. As shown in Figure 3I, all of the experimental groups demotrated obvious antibacterial activities in vitro against *S. aureus*, and the number of bacterial colonies forming units (CFUs) remarkably reduced when exposed to the hydrogel. A >97.5% killing ratio against *S. aureus* bacteria was achieved in the hydrogel groups (Figure 3J). In addition, CPO/PDANP, CPO/D@P and CPO/D@P/IGF‐1C showed an obvious antibacterial activity in vitro against *E. coli* with a >73.2% killing ratio. The CPO/D@P/IGF‐1C+NIR group showed significantly higher antibacterial activity against *E. coli* in vitro than that of the other groups due to the photothermal performance, and a 99.7% killing ratio against *E. coli* bacteria was achieved. The excellent antibacterial activity of the hydrogel was attributed to the protonated amino groups in CMCS and the Schiff base compounds generated in the hydrogel,^[^ ^30^ ^]^ and the combination of hydrogel with photothemarl can effectively enhance the antibacterial activity.

### 2.11 Biocompatibility and Promoted cell Migration of the Hydrogels

**Figure 4:**
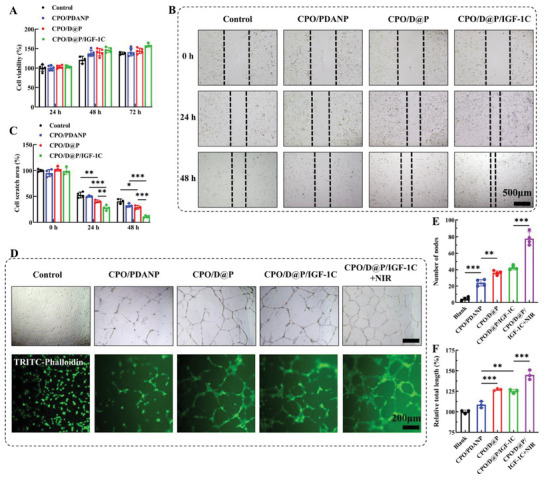
Hydrogels showed good biocompatibility, promoted cell migration and enhanced tube formation. A) Cell viability of HSF cells incubated with hydrogels, n = 5; B) Representative images of cell scratch experiments of different hydrogel groups; C) Quantification of cell‐free area in scratch experiments, n = 3; D) Representative images of angiogenesis experiments of different hydrogel groups, cells were stained with TRITC‐Phalloidin; Quantitative analysis of the number of nodes (n = 4) E) and relative total length (n = 3) F) in in vitro angiogenesis experiment. Tukey's multiple comparisons test, ^*^ *p* < 0.05, ^**^ *p* < 0.01, ^***^ *p* < 0.001.

The cell biocompatibility of the hydrogels was determined by CCK‐8 assay. Following incubation with hydrogel extracts for several days, all the hydrogel groups exhibited high cell viability and cell proliferation trends (**Figure** **4**A), indicating potent cell biocompatibility of the hydrogel system. CPO/D@P/IGF‐1C hydrogel group showed higher cell viability than the control group, because that IGF‐1C could promote cell proliferation.^[^ ^41^ ^]^

The effect of hydrogel on cell migration was evaluated by cell scratch assay. As shown in Figure 4B,C, cell migration was observed in all experimental groups and the denuded area was reduced with incubation time. Both CPO/D hydrogel and CPO/D@P/IGF‐1C hydrogel exhibited significant promotion in cell migration. After 24 h incubation, the denuded area was 52.8%, 48.7%, 40.5% and 28.2% for the control, CPO/PDANP, CPO/D@P and CPO/D@P/IGF‐1C, respectively. The denuded area reduced to 40.7%, 33.0%, 29.2% and 10.1% after 48 h for the control, CPO/PDANP, CPO/D@P and CPO/D@P/IGF‐1C, respectively. It has been reported that cell migration is integral to the wound repair process and dressings promoted cell migration in vitro could also enhance wound repair in vivo,^[^ ^30^, ^42^ ^]^ so the hydrogel system showed promising potential in diabetic wound healing.

### 2.12 In vitro Pro‐Angiogenesis Effect of the Hydrogels

The high glucose concentration in diabetic hampered angiogenesis, which impaired wound healing because blood vessels can provide nutrients for cells involved in healing and maintain the growth of newly‐formed granulation tissue,^[^ ^43^ ^]^ so hydrogel dressings with pro‐angiogenesis effect is particularly crucial for diabetic wound repair. In vitro tube formation assay was performed to assess the angiogenic activity of the hydrogels. Compared with the control and CPO/PDANP groups, CPO/D@P and CPO/D@P/IGF‐1C hydrogels exhibited a significant promotion in tube formation in vitro with a significant increase in tube length and junction formation (Figure 4D–F). The enhanced pro‐angiogenesis effect of CPO/D@P and CPO/D@P/IGF‐1C hydrogels contributed to DFO and IGF‐1C. Studies showed that DFO has been implicated as an angiogenic agent because of its ability to upregulate HIF‐1α and other key downstream angiogenic factors.^[^ ^39^ ^]^ IGF‐1C has been reported to play an important role in promoting angiogenesis in studies on wound healing, ischemia and acute myocardial infarction.^[^ ^44^, ^45^ ^]^ It is noted that the combination of CPO/D@P/IGF‐1C hydrogel and mild thermal stimulation (CPO/D@P/IGF‐1C+NIR group) was the most effective in promoting angiogenesis, with a markedly greater of nodes number and tube length. This may be caused by the synergistic effect of DFO, IGF‐1C and thermal stimulation, as studies have confirmed that mild thermal stimulation could stimulate the formation of endothelial cells.^[^ ^11^ ^]^

### 2.13 Diabetic Wound Healing and Blood Glucose Level Control In Vivo

**Figure 5:**
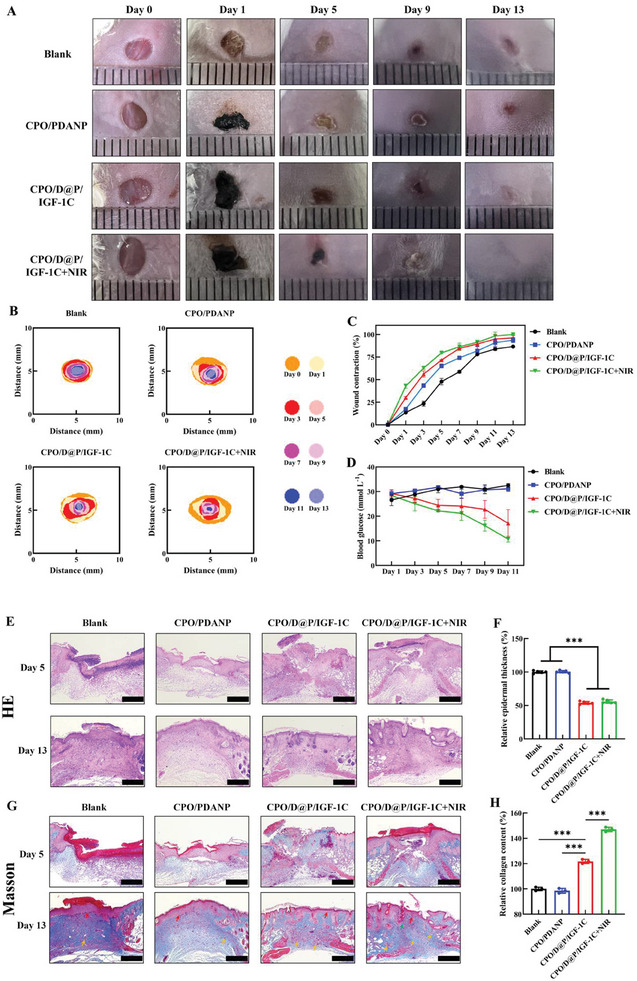
Hydrogels accelerated wound healing in diabetic mice. A) Representative image of wound healing; B) Wound closure traces during the therapy period; C) Wound healing rate curve; D) Blood glucose levels of mice during the wound healing process; E) Representative image of wound tissue with H&E staining; F) Relative epithelium thickness at the wound site on the 13th day, n = 3; G) Representative image of wound tissue with Masson trichrome staining (red arrows: boundary of epithelium and dermis, orange arrows: blood vessels, green arrows: hair follicles); H) Relative collagen content at the wound site on the 13th day, n = 3. The scale bar is 500 µm. Tukey's multiple comparisons test, ^***^ *p* < 0.001.

A full­thickness diabetic wound model was explored to verify the wound healing efficacy of the hydrogel system in vivo. As shown in **Figure** **5**A–C, the wounds of each group were photographed, the closure traces of each wound were traced, and the wound healing curves were diagrammed. The wound size of the hydrogel groups contracted faster than the other groups, and the average wound healing area after CPO/PDANP, CPO/D@P/IGF‐1C and CPO/D@P/IGF‐1C+NIR treatment was 43.8 ± 2.1%, 56.2 ± 3.4%, and 62.9 ± 1.3% after 3 days, respectively, which were significantly higher compared with the control group (23.5 ± 2.8%). The average wound healing area in CPO/PDANP, CPO/D@P/IGF‐1C and CPO/D@P/IGF‐1C+NIR groups increased to 74.1 ± 1.4%, 84.6 ± 0.4% and 84.6 ± 4.0% on the 7th day, respectively, which were larger than the control group (58.7 ± 1.6%). The wound in the CPO/D@P/IGF‐1C+NIR group was completely healed on the 13th day, while the average wound healing area in CPO/PDANP, CPO/D@P/IGF‐1C and control group was ≈93.7 ± 2.8%, 96.2 ± 2.8% and 86.5 ± 0.2%, respectively, indicating that the combination of CPO/D@P/IGF‐1C hydrogel dressing and mild thermal stimulation further promoted wound regeneration in vivo.

Studies have revealed that the high blood glucose levels resulting from diabetes impede wound healing because the excessive glucose deposition in blood can lead to peripheral neuropathy,^[^ ^46^ ^]^ inflammatory response disorder and accumulation of advanced glycation end products (AGEs),^[^ ^19^, ^47^, ^48^ ^]^ which has been confirmed to be associated with difficult diabetic wound healing. Thus, the management of blood glucose is favorable for wound healing in diabetes. The blood glucose levels of each mouse were monitored during the wound‐healing process. As shown in Figure 5D, the blood glucose levels in CPO/PDANP and control groups slowly elevated during the experimental period, while the blood glucose levels in CPO/D@P/IGF‐1C and CPO/D@P/IGF‐1C+NIR groups showed an obvious decreasing trend. The average blood glucose level descended to a much lower altitude on the 11th day, that's 17 and 11 mM for CPO/D@P/IGF‐1C and CPO/D@P/IGF‐1C+NIR treatment groups, respectively. The relatively lower blood glucose levels in CPO/D@P/IGF‐1C and CPO/D@P/IGF‐1C+NIR groups were due to the sustained release of IGF‐1C from the hydrogels, as IGF‐1C can regulate the glucose metabolism, promoting glucose uptake and utilization, and reducing blood sugar levels.^[^ ^13^ ^]^

### 2.14 Histomorphological Evaluation of Regenerated Skin Tissue

To further evaluate the quality of regenerated skin tissue, Masson's trichrome and H&E staining were performed. According to the H&E staining (Figure 5E), no obvious inflammatory response was found in the early stage of wound healing (day 5) for all the hydrogel groups, while more inflammatory cells with highly condensed chromatin can were observed in the wound sites for the control group. These results suggested that the CPO hydrogel system did not cause increased foreign body reaction. With the progress of the healing process, fewer inflammatory cells were found for all the groups on the 13th day. However, the control group still exhibited infiltration of inflammatory cells on the 13th day (Figure 5E), indicating that the wound was still in the inflammatory stage and had not yet entered the re‐epithelialization stage. Additionally, the epithelial thickness of the control and CPO/PDANP groups on the 13th day was remarkably thicker than those of CPO/D@P/IGF‐1C and CPO/D@P/IGF‐1C+NIR groups (Figure 5F), suggesting that the CPO/D@P/IGF‐1C and CPO/D@P/IGF‐1C+NIR groups had passed the inflammatory phase and completed re‐epithelization, as the process of re‐epithelization was characterized by the formation of mature thin epithelium from relatively thick epithelium.^[^ ^30^ ^]^ This may be due to the excellent antioxidant and anti‐inflammatory properties of the CPO/D@P/IGF‐1C hydrogel, which could promote the polarization of M2 macrophages.

Masson's trichrome staining (Figure 5G) showed that the collagen density in all groups increased from the 5th day to the 13th day, and the CPO/D@P/IGF‐1C+NIR group demonstrated the highest collage deposition (Figure 5H). Furthermore, CPO/D@P/IGF‐1C+NIR groups exhibited more blood vessels and hair follicles than other groups on the 13th day. Collagen deposition, vascularization and the formation of skin appendages like hair follicles are crucial for ECM remodeling and tissue regeneration during the wound repair process.^[^ ^1^ ^]^ Based on the results, the CPO/D@P/IGF‐1C+NIR group showed the most complete and mature skin structure. The reason was that the CPO/D@P/IGF‐1C hydrogel can improve wound healing by promoting cell migration and angiogenesis, and the combined therapy with mild heat stimulation can significantly promote skin cell proliferation and migration.^[^ ^11^ ^]^

### 2.15 Inflammation and Macrophages Polarization In Vivo

**Figure 6:**
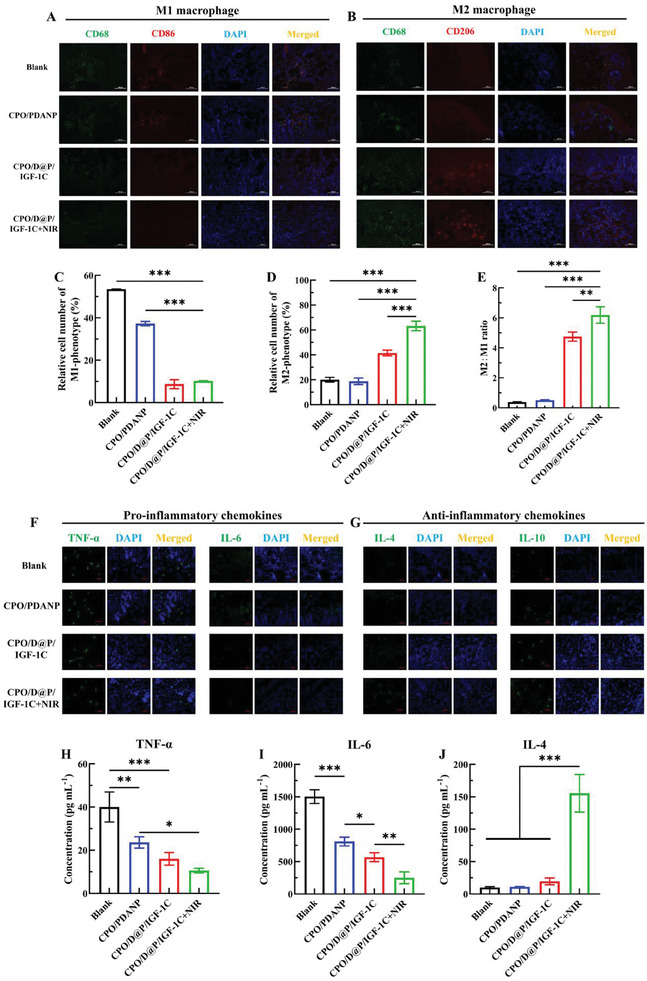
Hydrogels could modulate the inflammatory microenvironment in diabetic wounds by promoting the transformation of M1 to M2, down‐regulating the expression of pro‐inflammatory chemokines, and up‐regulating the expression of anti‐inflammatory chemokines. A) Immunofluorescence images of macrophages staining with macrophage phenotype markers in the regenerated tissues on day 5. Green: CD68 panmacrophages, red: CD86 M1 macrophages, Scale bar: 100 µm; B) Immunofluorescence images of macrophages staining with macrophage phenotype markers in the regenerated tissues on day 5. Green: CD68 panmacrophages, red: CD206 M2 macrophages, Scale bar: 100 µm; Quantification of M1 macrophage C) and M2 macrophage D) populations; E) The population ratio of M2/M1. F) Images of immunofluorescence staining with TNF‐α and IL‐6 (green) in the regenerated tissues on day 5. G) Images of immunofluorescence staining with IL‐4 and IL‐10 (green) in the regenerated tissues on day 5. The expression levels of TNF‐α H), IL‐6 I), and IL‐4 J) in the regenerated tissues on day 5 were tested by ELISA. Tukey's multiple comparisons test, n = 3, ^*^ *p* < 0.05, ^**^ *p* < 0.01, ^***^ *p* < 0.001.

Chronic inflammation is a hallmark of the diabetic wound microenvironment, which is an important factor that hinders the healing of diabetic wound.^[^ ^2^ ^]^ In the later stage of inflammation, M1 macrophages (pro‐inflammatory phenotype) transform into M2 macrophages (anti‐inflammatory phenotype), which promote the release of anti‐inflammatory factors and the production of ECM, and pave the wound for the proliferation and remodeling stages.^[^ ^49^ ^]^ In diabetic wounds, the transformation of macrophages from M1 to M2 phenotype is blocked, and wound healing usually stagnates in the inflammatory stage, preventing the progression to the proliferative stage, and impairing the wound repair.^[^ ^50^ ^]^ To assess the regulatory effect of different treatments on the macrophage phenotype, immunofluorescence staining of CD68/CD86 for M1 macrophages and CD68/CD206 for M2 macrophages was performed. It was shown that on the 5th day, the expression levels of M1 macrophages in the control and CPO/PDANP groups were relatively high, and the M2 macrophages were low (**Figure** **6**A,B). However, the expression levels of M1 macrophages were significantly low in the CPO/D@P/IGF‐1C and CPO/D@P/IGF‐1C+NIR groups, and the M2 macrophages were significantly high (Figure 6A,B). These results indicated that the wounds of the control and CPO/PDANP groups were still in the inflammatory stage, while the wounds in the CPO/D@P/IGF‐1C and CPO/D@P/IGF‐1C+NIR groups had entered into the next healing stage, which was consistent with the results of histomorphological evaluation. In addition, the CPO/D@P/IGF‐1C+NIR group showed the highest percentage of M2 macrophages and the population ratio of M2 to M1 macrophages (Figure 6C–E), indicating that the CPO/D@P/IGF‐1C+NIR treatment could reduce inflammatory reaction and improve the polarization of M2 macrophages. The result showed that the combination therapy of CPO/D@P/IGF‐1C and mild heat stimulation can promote the polarization of M2 macrophages, thus causing better anti‐inflammatory effects. This may be due to the antioxidant and anti‐inflammatory performance of the CPO/D@P/IGF‐1C, and the regulation effect of macrophage phenotype from M1 to M2 under mild heat stimulation.^[^ ^51^ ^]^

To further verify the healing stage transition, the inflammatory chemokines in wound tissues were analyzed by immunofluorescence staining. As shown in Figure 6F,G, the expression levels of TNF‐α and IL‐6 in CPO/D@P/IGF‐1C and CPO/D@P/IGF‐1C+NIR groups were partially reduced compared to those in the control and CPO/PDANP groups, and the expression levels of IL‐4 and IL‐10 in CPO/D@P/IGF‐1C and CPO/D@P/IGF‐1C +NIR groups were remarkably increased compared to those in the control and CPO/PDANP groups. Furthermore, the expression levels of inflammatory chemokines in regenerated tissues were tested by ELISA. As shown in Figure 6H–J, the pro‐inflammatory chemokines of TNF‐α and IL‐6 were much lower in the hydrogel groups compared with the control group. Although there was no remarkable increase of the anti‐inflammatory chemokine IL‐4 in CPO/PDANP and CPO/D@P/IGF‐1C groups, the expression level of IL‐4 in CPO/D@P/IGF‐1C+NIR group was much higher than other groups. These results demonstrated that CPO/D@P/IGF‐1C combined with mild heat could significantly shorten the inflammatory stage and promote diabetic wound healing by up‐regulating the expression of anti‐inflammatory factors and down‐regulating the expression of pro‐inflammatory factors.

### 2.16 Neovascularization in Regenerative Skin Tissue

**Figure 7:**
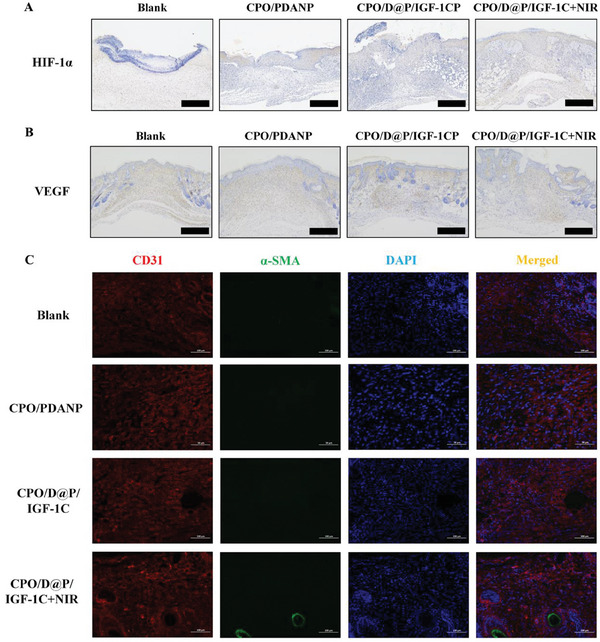
Hydrogels could enhance neovascularization in diabetic wounds by upregulating and angiogenesis‐related growth factors. A) Images of immunohistochemical staining with HIF‐1α in the regenerated tissues on day 5; B) Images of immunohistochemical staining with VEGF in the regenerated tissues on day 13; C) Images of immunofluorescence staining with CD31 (red) and α‐SMA (green) in the regenerated tissues on day 13. Scale bar: 500 µm in (A, B), 100 µm in (C).

Neovascularization is crucial for wound healing because blood vessels can provide nutrition for cells and maintain the growth of the newly‐formed granulation tissue. To evaluate the formation of new vessels at the wound sites, angiogenesis‐related growth factors were analyzed by immumohistochemical staining of HIF‐1α and VEGF and immunofluorescence staining of CD31 and α‐SMA. HIF‐1α and VEGF are key signaling molecules in the induction of angiogenesis.^[^ ^39^ ^]^ The expression of HIF‐1α is upregulated in hypoxic conditions, which subsequently upregulates the expression of VEGF and other factors.^[^ ^39^ ^]^ VEGF can act as a chemotactic agent to induce vascular permeability, endothelial cell migration and proliferation, thus promoting angiogenesis.^[^ ^39^ ^]^ As shown in **Figure** **7**A,B, the CPO/D@P/IGF‐1C and CPO/D@P/IGF‐1C+NIR group exhibited remarkedly higher expression levels of HIF‐1α and VEGF than the CPO/PDANP and CPO/D@P/IGF‐1C groups. The expression levels of HIF‐1α and VEGF in the CPO/D@P/IGF‐1C+NIR group were highest because DFO can promote HIF‐1α accumulation under normoxic and hypoxic conditions.^[^ ^39^ ^]^ CD31 and α‐SMA were vascular endothelial‐specific markers and vascular smooth muscle cell markers, respectively.^[^ ^30^ ^]^ CD31 immunohistochemical staining revealed a remarkably dense population of red CD31‐positive cells (red fluorescence) in the CPO/D@P/IGF‐1C and CPO/D@P/IGF‐1C+NIR groups compared to that in the control and CPO/PDANP groups (Figure 7C), indicating that dense microvascular structures were formed in the wounds of the CPO/D@P/IGF‐1C and CPO/D@P/IGF‐1C+NIR groups. Moreover, the green fluorescence of α‐SMA, which represents neonatally mature blood vessels is more pronounced in the CPO/D@P/IGF‐1C+NIR group (Figure 7C), confirming that angiogenesis in diabetic wounds was promoted by the combination of the CPO/D@P/IGF‐1C hydrogel and mild heat stimulation. Since the formation of neovascular could facilitate the nutrient supply and metabolic exchange of the wound tissues and have a significant effect on the restoration of skin tissue structure and function,^[^ ^52^ ^]^ the combined therapy of the CPO/D@P/IGF‐1C hydrogel and mild heat treatment can further accelerate the diabetic wound healing.

## 3 Conclusion

CPO/D@P/IGF‐1C hydrogel combined with mild thermal treatment was explored for diabetic wound healing. The CPO/D@P/IGF‐1C hydrogel exhibited good photothermal performance and sustained drug release. The hydrogel also showed excellent antioxidant, anti‐inflammatory and antibacterial activities, desirable mechanical and tissue adhesive performance, and good biocompatibility. Tube formation and scratch assays demonstrated that the CPO/D@P/IGF‐1C hydrogel could promote the tube formation of HUVECs and cell migration due to the drugs (DFO and IGF‐1C) released from the hydrogel. Moreover, the CPO/D@P/IGF‐1C hydrogel combined with mild heat treatment could promote diabetic wound healing in STZ‐induced diabetic wound mice and showed the most complete and mature skin structure with more collagen deposition, blood vessels and hair follicles. Immunofluorescence and immunohistochemistry results showed that the combination of CPO/D@P/IGF‐1C and mild heat stimulation could promote the expression levels of angiogenesis‐related growth factors including HIF‐1α, VEGF, CD31 and α‐SMA, and facilitate the transformation of macrophages from M1 phenotype to M2 phenotype, which in turn up‐regulate the expression levels of anti‐inflammatory factor IL‐4, and down‐regulate the expression levels of pro‐inflammatory factors TNF‐α and IL‐6. Thus, the combined treatment may offer a promising therapeutic strategy for diabetic wound healing.

## 4 Experimental Section

### Materials

Carboxymethyl chitosan (CMCS) was purchased from Bomeibio Co., Ltd. N‐Hydroxysuccinimide (NHS) was purchased from Yuanye Biotechnology (Shanghai) Co., Ltd. Dextran, sodium periodate (NaIO_4_), dopamine hydrochloride (DA), 1,3,5‐trimethyl benzene (TMB), triethylamine (TEA) and deferoxamine mesylate (DFO) were purchased from Aladdin (Shanghai) Co., Ltd. Glycerol was purchased from Sinopharm (China) Co., Ltd. Pluronic F127 was purchased from Beyotime Co., Ltd. IGF‐1C was purchased from Synthbio Co., Ltd. 4‐nitrophenyl 4‐(4,4,5,5‐tetramethyl‐1,3,2‐dioxaborolan‐2‐yl) benzyl carbonate (NBC) was synthesized according to this previous work.^[^ ^20^ ^]^ All the reagents were used as received without further purification.

### Synthesis of CMCS‐PBA

CMCS‐PBA was synthesized according to the previous report.^[^ ^53^ ^]^ Briefly, 107 mg of CMCS (0.5 mmol) was dissolved in 15 mL of deionized water, and 0.3 mL of TEA was added. The mixture was stirred at 25 °C for 30 min. Then, 200 mg of NBC (0.5 mmol) and 73 mg of NHS (0.6 mmol) dissolved in 15 mL of tetrahydrofuran (THF) was added to the CMCS/TEA solution, and the mixture was reacted at 25 °C for 12 h. The mixture was transferred into a dialysis tube (MWCO 8000–14000) and dialyzed against 5% methanol aqueous solution for one day, and then against deionized water for two days with four changes every day. The residue was freeze‐dried to obtain a white cotton‐like product of CMCS‐PBA with a yield of 51.5%.

### Synthesis of OXD

The synthesis of OXD was referred to in the previous report.^[^ ^54^ ^]^ Briefly, 1000 mg of dextran was dissolved in 30 mL of deionized water at 25 °C under stirring. 600 mg of NaIO_4_ (2.8 mmol) dissolved in 20 mL of deionized water was slowly added to the dextran solution, and the mixture was reacted at 25 °C for 24 h. Then, 600 mg of glycerol (6.5 mmol) dissolved in 10 mL of deionized water was added slowly under stirring, and the mixture was reacted at 25 °C for another 30 min. After that, the mixture was transferred into a dialysis tube (MWCO 8000–14000) and dialyzed against deionized water for three days with four changes every day. The residue was freeze‐dried to obtain a white cotton‐like product of OXD with a yield of 93.0%. All the process was carried out in the dark.

### Preparation of Polydopamine Nanoparticles (PDANP) and DFO‐Loaded Polydopamine Nanoparticles (D@P)

The preparation of PDANP was referred to as the previously reported method.^[^ ^11^ ^]^ Briefly, 360 mg of Pluronic F127 and 417 µL of TMB were dissolved in 60 mL of deionized water, and 60 mL of ethanol was added under stirring at 25 °C for 30 min. Then, 60 mg of DA and 90 mg of Tris were added to the above solution and stirred at 25 °C for 24 h. Next, the reaction mixture was added to eight times the volume of acetone under a quick stir. After standing for 30 min, the mixture was centrifuged at 3000 r min^−1^ for 5 min, the precipitation was collected and then washed with acetone 3 times to obtain the product of PDANP.

20 mg of PDANP was dispersed in 4 mL of deionized water, and the pH of the solution was adjusted to 7.4 using 2 M NaOH. 14.4 mg of DFO dissolved in 1 mL of deionized water was slowly added into the PDANP dispersion, and the mixture was stirred at 25 °C for 12 h. Next, the mixture was added to eight times the volume of acetone under a quick stir. After standing for 30 min, the mixture was centrifuged at 3000 r min^−1^ for 5 min, the precipitation was collected and then washed with acetone 3 times to obtain the product of D@P. The drug loading efficiency (DLE) and drug encapsulation efficiency (DEE) were calculated using Equations (1) and (2), respectively.

(1) $$DLE\;=\;\frac{m_{1}}{m_{2}}\times 100\%$$

(2) $$DEE\;=\;\frac{m_{1}}{m_{3}}\times 100\%$$

where *m_1_* is the mass of DFO loaded in D@P, *m_2_* is the mass of D@P, and *m_3_* is the feeding mass of DFO.

### Preparation of CMCS‐PBA‐OXD Hydrogel (CPO) and IGF‐1C and D@P‐Loaded Hydrogel (CPO/D@P/IGF‐1C)

CMCS‐PBA and OXD were dissolved in deionized water at 4 wt.%, respectively. The pH value of the CMCS‐PBA solution was adjusted to 8.0 using 2 m NaOH. CMCS‐PBA and OXD solution were mixed in equal volume and stirred to form a uniform mixture, and CPO hydrogel was formed after ≈30 s.

For drug‐loaded hydrogel, D@P and/or IGF‐1C were added into the OXD solution to form a uniform mixture. Then, CMCS‐PBA solution was mixed with OXD/D@P/IGF‐1C solution in equal volume under stirring, and CPO/D@P/IGF‐1C hydrogel was formed after ≈30 s.

### Characterization

The chemical structure of samples was analyzed using ^1^H NMR (Bruker AVANCE III 400 MHz) and FTIR (Nicolet 6700, Thermo Nicolet, USA). The size distribution of PDANP and D@P was tested by dynamic light scattering (DLS) on a Zetasizer (Nano Series, Malvern Instruments, UK), and the morphology of nanoparticles was observed under a transmission electron microscope (TEM, HITACHI HT‐7800). The microstructure of hydrogel was observed under a scanning electron microscope (Hitachi S‐4300, Nova NanoSEM 450, FEI, USA). Hydrogels were prepared and freeze‐dried, and morphology in the cross‐section was observed. The photo‐thermal performance of nanoparticles and hydrogels was tested. Nanoparticles were dispersed in PBS at 1 mg mL^−1^. 3 mL of the dispersion in cuvette was irradiated under an 808 nm NIR laser (1 W, 10 min, 15 cm), and the temperature of the solution was monitored using a Forward Looking Infra‐Red (FLIR) thermal imaging system. 1 mL of CPO/D@P hydrogels with different content of D@P were placed on a plate and irradiated under an 808 nm NIR laser (1W, 10 min, 15 cm), and the temperature of the hydrogels was recorded.

### Rheological Analysis

The rheological properties of hydrogels were tested using a rotating rheometer (TA, HAAKE Mars III). 0.1 mL of the prepared CMCS‐PBA solution and OXD solution were transferred to a 25 mm parallel plate, and after rapid mixing, time scanning was performed at 37 °C to monitor the elastic modulus (G′) and loss modulus (G″) of the hydrogels. The gap was set at 0.2 mm and the frequency was 10 rad s^−1^. The strain amplitude was set at 2% to ensure a linear viscoelastic region. Frequency scanning was performed to monitor the G′, G″ and viscosity (η) of the hydrogels when the modulus reached a stable plateau. The frequency scan ranges from 0.1 to 100 rad s^−1^.

### Self‐Healing and Injectable Properties of Hydrogels

Two pieces of the as‐prepared CPO hydrogels (a small amount of methylene blue or phenol red was added for staining) were placed closely, and the self‐healing performance of the hydrogels was observed and recorded using a camera after 5 min.

CMCS‐PBA solution was mixed with an equal volume of OXD solution and quickly transferred to a syringe (1 mL, 0.45 mm). After 5 min, the mixture was extruded through the syringe to evaluate the injectable property of the hydrogel.

### Swelling and Degradation Properties of Hydrogels

The as‐prepared hydrogels were immersed in 10 mL of PBS (pH 7.4, 0.01 m) and incubated at 37 °C. At regular intervals, the hydrogels were taken out and weighed after absorbing the surface water with filter paper. The swelling ratio of the hydrogel was calculated using Equation (3):

(3) $$Swelling\;ratio\;\left(\%\right)=\frac{M_{t}}{M_{0}}\;*100$$

where *M_t_* is the mass of the hydrogel at time *t*, and *M_0_* is the initial mass of the hydrogel.

The degradation behavior of the hydrogels was tested when the hydrogels reached swelling equilibrium. Hydrogels were immersed in 10 mL of PBS (pH = 7.4, 0.01 m) and incubated at 37 °C. At regular intervals, the hydrogels were taken out and weighed after absorbing the surface water with filter paper. The remaining ratio of the hydrogel was calculated using Equation (4):

(4) $$Remaining\;ratio\;\left(\%\right)=\frac{M_{r}}{M_{e}}\;*100$$

where *M_e_* is the mass of the hydrogel at swelling equilibrium, and *M_r_* is the remaining mass of the hydrogel at a specific time.

### Drug Release of Hydrogels

CPO/D@P/IGF‐1C hydrogels (1 mL) were put in brown vials containing 10 mL of PBS with different pH, H_2_O_2_ and glucose concentrations. The vials were incubated at 37 °C. At regular intervals, 2 mL of media was withdrawn and replenished with 2 mL of fresh media to keep the total volume of the medium constant. The amount of released DFO was determined by HPLC at 214 nm using a Supersil ODS2 E321498 column (200 mm × 4.6 mm, 5.0 µm) at 25 °C. The mobile phase was acetonitrile/PBS (v/v, 15/85) and the flow rate was 1 mL min^−1^. The amount of IGF‐1C was determined by HPLC at 220 nm using a YMC‐Pack Protein‐RP column (150 mm × 4.6 mm, 5.0 µm, Agilent) at 25 °C. The mobile phase was acetonitrile/water (v/v, 4/96) containing 0.1% trifluoroacetic acid and the flow rate was 1 mL min^−1^.

### Mechanical and Adhesive Properties of the Hydrogels

The mechanical properties of the hydrogels were evaluated by tensile and compression testing. The adhesive properties of the hydrogels were macroscopic qualitatively evaluated by a visceral adhesion test in mice skin adhesion under torsion stress and joint adhesion and quantitatively evaluated by testing the adhesive strengths of the hydrogels on mice skin.

### Biocompatibility of Hydrogel

A CCK‐8 assay kit was employed to evaluate the cytocompatibility of the hydrogels. Briefly, human skin fibroblasts (HSFs) were seeded at 1 × 10^4^ cells/well in a 96‐well plate. After 24 h incubation, the DMEM was replaced with 100 µL of hydrogel PBS extract (1 mL hydrogel incubated in 10 mL PBS for 72 h) and incubated for another period of time. Then, the medium was replaced with 100 µL of DMEM containing 10% CCK‐8 and the absorbance of each well was recorded at 450 nm using a microplate reader (Putian, Beijing) after 2 h incubation. Cell viability was calculated using Equation (5):

(5) $$Cell\;viability\;\;\left(\%\right)=\left(Int_{e}-Int_{b})/(Int_{c}-Int_{b}\right)\times 100\%$$

where *Int*_e_, *Int*_b_ and *Int*_c_ represent the absorption intensity of the cells cocultured with hydrogel extract (experimental group), culture medium only (blank group) and cells cultured with medium (control group), respectively.

### Antioxidant Property of Hydrogels

The antioxidant activity of hydrogels was evaluated by scavenging DPPH and ABTS^+•^ free radical and intracellular ROS. DPPH free radical scavenging assay was performed according to the previous report.^[^ ^55^ ^]^ Briefly, the prepared hydrogel sample was added into the 5 fold volume of DPPH methanol solution (75 µM) and the mixture was incubated at 37 °C in the dark for 30 min. Then, the absorbance of the DPPH solution was recorded at 517 nm using a microplate reader (Putian, Beijing). DPPH scavenging activity was calculated using Equation (6):

(6) $$\mathrm{DPPH}\;\mathrm{scavenging}\;\mathrm{activity}\;(\%)=(1-\frac{Abs_{s}-Abs_{m}}{Abs_{D}})\times 100$$

where *Abs_s_* is the absorbance of the sample, *Abs_m_* is the absorbance of methanol, and *Abs_D_* is the absorbance of the DPPH solution.

ABTS^+•^ free radical scavenging assay was performed according to the previous report.^[^ ^56^ ^]^ Briefly, the prepared hydrogel sample was added to the 10 fold volume of ABTS^+•^ solution and incubated at 37 °C in the dark. The absorbance of the mixture was recorded at 734 nm using a microplate reader (Putian, Beijing). ABTS^+•^ scavenging activity was calculated using Equation (7):

(7) $$\mathrm{ABT}S^{+•}\;\mathrm{scavenging}\;\mathrm{activity}\;\left(\%\right)=\left(1-\frac{Abs_{s}-Abs_{P}}{Abs_{A}}\right)\times 100$$

where *Abs_s_* is the absorbance of the sample, *Abs_P_* is the absorbance of PBS, and *Abs_A_* is the absorbance of ABTS^+•^ solution.

The intracellular ROS scavenging ability of hydrogel was measured by reactive oxygen species assay (Beyotime Biotechnology, Shanghai, China) using 2′,7′‐dichlorofluorescein diacetate (DCFH‐DA) as a fluorescence probe.^[^ ^57^ ^]^ HSFs were seeded in a 24‐well plate at a density of 5 × 10^5^ cells per well and incubated for 12 h. The cells were first treated with 2 mL DMEM containing 100 µM H_2_O_2_ for 2 h and then incubated with 2 mL hydrogel PBS extract (1 mL hydrogel incubated in 10 mL PBS for 48 h) for 24 h. Subsequently, the cells were washed with PBS thrice and then treated with 2 mL DMEM containing DCFH‐DA (10 µM in serum‐free medium) for 20 min. After that, the cells were washed with PBS again and observed under a fluorescence microscope (CKX53, Olympus) excited at 488 nm.

### Anti‐Inflammatory Property of Hydrogels

The in vitro anti‐inflammatory activity of the hydrogels was tested. Raw 264.7 cells were seeded in a 24‐well plate at 5 × 10^5^ cells/well in 2 mL of complete DMEM and incubated for 12 h. Then, the cells were treated with 2 mL DMEM containing LPS (1 µg mL^−1^ in serum‐free medium). After cultivation for 24 h, the cells were washed with PBS thrice and then treated with 2 mL hydrogel extract (1 mL hydrogel incubated in 10 mL DMEM for 48 h). After 72 h incubation, the concentrations of pro‐inflammatory cytokines (IL‐6 and TNF‐α) and anti‐inflammatory cytokines (IL‐4 and IL‐10) in the cell culture supernatant were measured using ELISA kits.

### Antibacterial activity of the hydrogels

In vitro antibacterial activities of the hydrogels were tested using *Escherichia coli* (*E. coli*, 25922) and *Staphylococcus aureus* (*S. aureus* 6538). 150 µL of the sterilized CPO, CPO/PDANP, CPO/D@P and CPO/D@P/IGF‐1C hydrogels were injected into the 96‐well plates, respectively. The hydrogels were immediately gelled and covered the bottom of the plate. Then, 150 µL of bacterial suspension (10^5^ CFU mL^−1^) was added to the well plate and incubated for 3 h. The CPO/D@P/IGF‐1C+NIR group was irradiated by an 808 nm NIR laser (1 W, 10 min). After resuspending the bacterial survivors, the diluted bacterial suspension was added to the solid medium and cultured for 18 h. Each set of tests was repeated five times. The CFU on the solid medium was counted. The killing ratio of bacteria was expressed using Equation (8):

(8) $$\mathrm{Kill}\;\;\left(\%\right)=\left(C_{s}-C_{h}\right)/C_{s}\times 100\%$$

*C_S_*: Cell count of control survivor, *C_h_*: cell count on hydrogels

### Tube Formation Assay

Matrigel (Solarbio, Beijing) was added in the lower chamber of the Transwell (150 µL per well) and incubated at 37 °C for 30 min to form a hydrogel. Human umbilical vein endothelial cells (HUVECs) were seeded on the hydrogel surface at a density of 5 × 10^5^ cells/well and incubated in complete DMEM. Then, 100 µL of various hydrogels were placed in the upper chamber of the plate (incubated in 200 µL PBS), with or without NIR irradiation (1 W cm^−2^) for 10 min. After 4 h of incubation, the cells were subsequently stained with TRITC‐Phalloidin for 20 min and observed under an inverted fluorescence microscope, and the tube formation was analyzed by the angiogenesis analysis plugin of Image J software.

### Cell Scratch Assay

HSFs were seeded in a 24‐well plate at a density of 5 × 10^5^ per well and incubated in 500 µL of DMEM for 24 h to form a single layer of cells at the bottom of the plates. Then, wounds were made by scratching the cell layer using a 10 µL pipette tip. Subsequently, the medium was removed and the cells were washed gently with PBS three times to remove the cell debris. After that, the cells were incubated with hydrogel extract (1 mL hydrogel incubated in 10 mL DMEM for 72 h) for several days. The wounds in identical locations were observed under an inverted microscope. Cell scratch area was calculated using Equation (9):

(9) $$\mathrm{Cell}\;\mathrm{scratch}\;\mathrm{area}\;=\frac{S_{t}}{S_{0}}\;\times 100\%$$

where *S_0_* is the scratch area at 0 h and *S_t_* is the scratch area at time *t*.

### Evaluation of Diabetic Wound Healing in Mice

Diabetic wound models were established on the back of BALB/c mice (female, 6–8 weeks, 20–25 g). All animal experiments were approved by the Animal Ethics Committee of Ningbo University (Certificate number: NBU20230159). Type 1 diabetes models were established through five consecutive days of intraperitoneal injection of STZ (50 mg kg^−1^). The model was successfully established when the blood glucose levels of mice were higher than 16.7 mmol L^−1^ for two consecutive weeks. Full‐thickness skin defect models were made on the back of mice with a needle biopsy instrument (4 mm in diameter). The diabetic mice were randomly divided into 4 groups (n = 6), namely, the control, CPO/PDANP, CPO/D@P/IGF‐1C, and CPO/D@P/IGF‐1C+NIR groups. The CPO/D@P/IGF‐1C+NIR group was irradiated by an 808 nm NIR laser (1 W, 15 cm, 10 min) for five consecutive days. The blood glucose level and wound of each mouse were monitored every other day. Wound area was measured using Image J software and the wound contraction was calculated using Equation (10):

(10) $$Wound\;contraction\;\left(\%\right)=\left(A_{0}-A_{t}\right)/A_{0}\times 100$$

where *A_0_* is the wound area at day 0, and *A_t_* is the wound area on the indicated day.

### Histopathological, Immunofluorescence and Immunohistochemical Analysis

At predetermined time points, two mice in each group were sacrificed. Tissues around the wound area were removed and fixed in 4% paraformaldehyde, and then embedded in paraffin and sliced at a thickness of 4 µm. The slices were stained with hematoxylin/eosin (H&E) and Masson's trichrome (MT) for histopathological analysis. Hypoxia‐inducible factor‐1α (HIF‐1α), vascular endothelial growth factor (VEGF), neovascularization (CD31 and α‐SMA), panmacrophage markers (CD68), M1 macrophage markers (CD86), M2 macrophage markers (CD206) were observed and quantitatively analyzed.

### Evaluation of Cytokines in Wound Tissues

Immunofluorescence staining of IL‐6, TNF‐α, IL‐10 and IL‐4 was used to evaluate the expression of inflammatory chemokines in the regenerated tissues. In addition, the expression levels of inflammatory chemokines were quantitatively analyzed by ELISA. In brief, tissues around the wound area were removed, rinsed with pre‐cooled PBS and cut into pieces after weighing. Then, the tissue pieces were grinded with an appropriate amount of pre‐cooled PBS solution (with PMSF) by a glass homogenizer. The homogenate was centrifuged at 10 000 × g for 15 min under 4 °C and the supernatant was collected for cytokines measurement. IL‐6, TNF‐α, and IL‐4 were determined by ELISA kit under the standard procedure.

### Statistical Analysis

The analysis data were presented as the mean  ±  standard deviation (n≥3), ^*^ *p*<0.05, ^**^ *p*<0.01, ^***^ *p*<0.001. One‐way or two‐way ANOVA with Tukey's multiple comparisons test was used to analyze the statistical significance by GraphPad Prism.

## Conflict of Interest

The authors declare no conflict of interest.

## Author Contributions

F.D. performed data curation, formal analysis, methodology, and wrote the final manuscript. J.Z. performed formal analysis, methodology, and investigation. F.C. performed formal analysis, methodology, and investigation. X.C. Performed investigation, and resources. H.L. performed supervision, validation, and visualization. C.J.L. performed visualization, investigation, and resources. L.Z. performed conceptualization, methodology, funding acquisition, and wrote, reviewed, and edited the final manuscript. H.T. performed funding acquisition, wrote, reviewed, and edited the final manuscript, and visualization.

## Supporting information



Supporting Information

## Data Availability

The data that support the findings of this study are available from the corresponding author upon reasonable request.

## References

[1] J. Liu , M. Qu , C. Wang , Y. Xue , H. Huang , Q. Chen , W. Sun , X. Zhou , G. Xu , X. Jiang , Small 2022, 18, 2106172. 10.1002/smll.202106172 35319815

[2] H. S. Kim , X. Sun , J. H. Lee , H. W. Kim , X. Fu , K. W. Leong , Adv. Drug. Deliver. Rev. 2019, 146, 209. 10.1016/j.addr.2018.12.014 30605737

[3] S. Matoori , A. Veves , D. J. Mooney , Sci. Transl. Med. 2021, 13, eabe4839. 33731435 10.1126/scitranslmed.abe4839

[4] Y. Fu , Y. Shi , L. Wang , Y. F. Zhao , R. K. Wang , K. Li , S. T. Zhang , X. J. Zha , W. Wang , X. Zhao , W. Yang , Adv. Sci. 2023, 10, 2206771. 10.1002/advs.202206771 PMC10161050 36862027

[5] F. Huang , X. Lu , Y. Yang , Y. Yang , Adv. Sci. 2023, 10, 2203308. 10.1002/advs.202203308 PMC9839871 36424137

[6] P. Gkogkolou , M. Bohm , Dermato‐Endocrinology 2012, 4, 259. 23467327 10.4161/derm.22028 PMC3583887

[7] R. Ramasamy , S. F. Yan , A. M. Schmidt , N. Y. Annals , Acad. Sci. 2011, 1243, 88. 10.1111/j.1749-6632.2011.06320.x PMC4501013 22211895

[8] H. Wang , Z. Xu , M. Zhao , G. Liu , J. Wu , Biomater. Sci. 2021, 9, 1530. 33433534 10.1039/d0bm01747g

[9] R. Yang , W. Xue , H. Liao , F. Wu , H. Guo , W. Zhang , P. Wang , X. Tan , H. Xu , B. Chi , Int. J Biol. Macromol. 2022, 223, 950. 36375676 10.1016/j.ijbiomac.2022.11.065

[10] L. Zhao , L. Niu , H. Liang , H. Tan , C. Liu , F. Zhu , ACS Appl. Mater. Interfaces. 2017, 9, 37563. 28994281 10.1021/acsami.7b09395

[11] Y. Yuan , D. Fan , S. Shen , X. Ma , Chem. Eng. J. 2022, 433, 133859.

[12] M. Jansen , F. M. van Schaik , A. T. Ricker , B. Bullock , D. E. Woods , K. H. Gabbay , A. L. Nussbaum , J. S. Sussenbach , J. L. Van den Brande , Nature 1983, 306, 609. 6358902 10.1038/306609a0

[13] A. Kasprzak , Int. J. Mol. Sci. 2021, 22, 6434.34208601

[14] S. Sukhanov , Y. Higashi , S. Y. Shai , C. Vaughn , J. Mohler , Y. Li , Y. H. Song , J. Titterington , P. Delafontaine , Arterioscl. Throm. Vas. 2007, 27, 2684. 10.1161/ATVBAHA.107.156257 17916769

[15] N. Yamada , R. Yanai , M. Nakamura , M. Inui , T. Nishida , Invest. Ophth. Vis. Sci. 2004, 45, 1125. 10.1167/iovs.03-0626 15037578

[16] T. Sun , X. Guo , R. Zhong , C. Wang , H. Liu , H. Li , L. Ma , J. Guan , C. You , M. Tian , Front. Bioeng. Biotech. 2020, 8, 53. 10.3389/fbioe.2020.00053 PMC7026261 32117933

[17] B. Tao , C. Lin , Z. Yuan , Y. He , M. Chen , K. Li , J. Hu , Y. Yang , Z. Xia , K. Cai , Chem. Eng. J. 2021, 403, 126182.

[18] L. Sheng , Z. Zhang , Y. Zhang , E. Wang , B. Ma , Q. Xu , L. Ma , M. Zhang , G. Pei , J. Chang , Biomaterials 2021, 264, 120414. 32980635 10.1016/j.biomaterials.2020.120414

[19] K. Maruyama , J. Asai , M. Li , T. Thorne , Am. J. Pathol. 2007, 170, 1178. 17392158 10.2353/ajpath.2007.060018 PMC1829452

[20] F. Dai , F. Chen , J. Zhang , X. Chen , H. Liang , Z. Liang , S. Zhang , H. Tan , L. Zhao , ACS Appl. Nano. Mater. 2024, 7, 7289.

[21] J. Maia , L. Ferreira , R. Carvalho , M. A. Ramos , M. H. Gil , Polymer 2005, 46, 9604.

[22] Z. Yuan , C. Lin , Y. He , B. Tao , M. Chen , J. Zhang , P. Liu , K. Cai , ACS Nano 2020, 14, 3546. 32069025 10.1021/acsnano.9b09871

[23] T. Elshaarani , H. Yu , L. Wang , J. Feng , C. Li , W. Zhou , A. Khan , M. Usman , B. Ul Amin , R. Khan , Int. J. Biol. Macromol. 2020, 161, 109. 32512091 10.1016/j.ijbiomac.2020.06.012

[24] J. Zhang , J. Xu , J. Lim , J. K. Nolan , H. Lee , C. H. Lee , Adv. Healthcare Mater. 2021, 10, 2100194. 10.1002/adhm.202100194 33930258

[25] C. Ding , L. Zhao , F. Liu , J. Cheng , J. Gu , D. Shan , C. Liu , X. Qu , Z. Yang , Biomacromolecules 2010, 11, 1043. 20337439 10.1021/bm1000179

[26] Y. Liang , M. Li , Y. Yang , L. Qiao , H. Xu , B. Guo , ACS Nano 2022, 16, 3194. 35099927 10.1021/acsnano.1c11040

[27] C. Tong , X. Zhong , Y. Yang , X. Liu , G. Zhong , C. Xiao , B. Liu , W. Wang , X. Yang , Biomaterials 2020, 243, 119936. 32171103 10.1016/j.biomaterials.2020.119936

[28] J. Zhang , F. Chen , D. Yu , Z. Liang , F. Dai , H. Liang , H. Li , H. Tan , L. Zhao , Int. J. Pharmaceut. 2023, 643, 123246. 10.1016/j.ijpharm.2023.123246 37467814

[29] D. Li , K. Chen , H. Tang , S. Hu , Adv. Mater. 2022, 34, 2108430. 10.1002/adma.202108430 34921569

[30] Y. Yuan , S. Shen , D. Fan , Biomaterials 2021, 276, 120838. 34274780 10.1016/j.biomaterials.2021.120838

[31] L. Wang , X. Zhang , K. Yang , Y. V. Fu , Adv. Funct. Mater. 2020, 30, 1904156.

[32] S. Usuki , Y. Y. Tsai , K. Morikawa , S. Nonaka , Y. Okuhara , M. Kise , R. K. Yu , PLoS One 2011, 6, e28693. 22194889 10.1371/journal.pone.0028693 PMC3237479

[33] S. F. Elhabal , N. Abdelaal , S. A. K. S. Al‐Zuhairy , M. F. M. Elrefai , A. M. E. Hamdan , M. M. Khalifa , S. Hababeh , M. A. Khasawneh , G. M. Khamis , J. Nelson , P. M. Mohie , R. A. Gad , A. Rizk , S. L. Kabil , M. K. El‐Ashery , B. R. Jasti , N. A. Elzohairy , T. Elnawawy , F. E. Hassan , M. El‐Nabarawi , Int. J. Nanomedicine. 2024, 19, 3045. 38559447 10.2147/IJN.S455270 PMC10981898

[34] B. Z. Johnson , A. W. Stevenson , C. M. Prele , M. W. Fear , F. M. Wood , Biomedicines 2020, 8, 101. 32365896 10.3390/biomedicines8050101 PMC7277690

[35] W. Ouyang , A. O'Garra , Immunity 2019, 50, 871. 30995504 10.1016/j.immuni.2019.03.020

[36] G. R. Ko , J. S. Lee , Tissue. Eng. Regen. Med. 2022, 19, 221. 35041181 10.1007/s13770-021-00419-z PMC8971302

[37] Y. Wang , M. Deng , Y. Wu , C. Hu , B. Zhang , C. Guo , H. Song , Q. Kong , Y. Wang , Compos. Part. B‐Eng. 2022, 236, 109806.

[38] Z. Tu , M. Chen , M. Wang , Z. Shao , X. Jiang , K. Wang , Z. Yao , S. Yang , X. Zhang , W. Gao , C. Lin , B. Lei , C. Mao , Adv. Funct. Mater. 2021, 31, 2100924.

[39] P. Holden , L. S. Nair , Tissue. Eng. Part. B‐Re. 2019, 25, 461. 10.1089/ten.TEB.2019.0111 31184273

[40] X. Cao , L. Duan , H. Hou , Y. Liu , S. Chen , S. Zhang , Y. Liu , C. Wang , X. Qi , N. Liu , Z. Han , D. Zhang , Z. C. Han , Z. Guo , Q. Zhao , Z. Li , Theranostics 2020, 10, 7697. 32685014 10.7150/thno.45434 PMC7359093

[41] G. Feng , J. Zhang , Y. Li , Nie, D. Z. , R. Wang , J. Liu , J. Gao , N. Liu , N. He , W. Du , H. Tao , Y. Che , Y. Xu , D. Kong , Q. Zhao , Z. Li , J. Am. Soc. Nephrol. 2016, 27, 2357. 26869006 10.1681/ASN.2015050578 PMC4978042

[42] M. Deng , Y. Wu , Y. Ren , H. Song , L. Zheng , G. Lin , X. Wen , Y. Tao , Q. Kong , Y. Wang , J. Control. Release. 2022, 350, 613. 36058354 10.1016/j.jconrel.2022.08.053

[43] X. Zhao , D. Pei , Y. Yang , K. Xu , J. Yu , Y. Zhang , Q. Zhang , G. He , Y. Zhang , A. Li , Y. Cheng , X. Chen , Adv. Funct. Mater. 2021, 31, 2009442.

[44] Q. Li , J. Cui , H. Huang , Z. Yue , Y. Chang , N. Li , Z. Han , Z. C. Han , Z. Guo , Z. Li , Future. Med. Chem. 2020, 12, 1239. 32351127 10.4155/fmc-2020-0071

[45] N. Zhao , Z. Yue , J. Cui , Y. Yao , X. Song , B. Cui , X. Qi , Z. Han , Z. C. Han , Z. Guo , Z. X. He , Z. Li , Stem. Cell. Res. Ther. 2019, 10, 129. 31036073 10.1186/s13287-019-1230-0 PMC6489284

[46] G. Theocharidis , A. Veves , Auton. Neurosci‐Basic. 2020, 223, 102610. 10.1016/j.autneu.2019.102610 PMC6957730 31790954

[47] M. Khan , H. Liu , J. Wang , B. Sun , Food. Res. Int. 2020, 130, 108933. 32156381 10.1016/j.foodres.2019.108933

[48] Q. Wang , X. Cao , G. Zhu , T. Xie , K. Ge , Y. Niu , Int. J. Diabetes. Dev. C. 2020, 40, 283.

[49] D. Skuratovskaia , M. Vulf , O. Khaziakhmatova , V. Malashchenko , A. Komar , E. Shunkin , V. Shupletsova , A. Goncharov , O. Urazova , L. Litvinova , Biomedicines 2020, 8, 400. 33050138 10.3390/biomedicines8100400 PMC7600904

[50] T. Umehara , R. Mori , K. A. Mace , T. Murase , Y. Abe , T. Yamamoto , K. Ikematsu , Diabetes 2019, 68, 617. 30523028 10.2337/db18-0313

[51] J. Zhang , Y. Wang , N. Ding , P. Ma , Z. Zhang , Y. Liu , Colloid. Interfac. Sci. 2023, 56, 100733.

[52] D. He , X. Liu , J. Jia , B. Peng , N. Xu , Q. Zhang , S. Wang , L. Li , M. Liu , Y. Huang , X. Zhang , Y. Yu , G. Luo , Adv. Funct. Mater. 2023, 34, 2306357.

[53] C. Shen , L. Zhao , X. Du , J. Tian , Y. Yuan , M. Jia , Y. He , R. Zeng , R. Qiao , C. Li , Mol. Pharmaceutics 2021, 18, 1419. 10.1021/acs.molpharmaceut.0c01245 33522827

[54] M. C. Giano , Z. Ibrahim , S. H. Medina , K. A. Sarhane , J. M. Christensen , Y. Yamada , G. Brandacher , J. P. Schneider , Nat. Commun. 2014, 5, 4095. 24958189 10.1038/ncomms5095 PMC4096704

[55] W. C. Liu , H. Y. Wang , T. H. Lee , R. J. Chung , Mat. Sci. Eng. C‐mater. 2019, 101, 630. 10.1016/j.msec.2019.04.018 31029356

[56] H. Zheng , H. Li , H. Deng , W. Fang , X. Huang , J. Qiao , Y. Tong , Colloids. Surf. B‐biointerfaces. 2022, 214, 112433. 35278858 10.1016/j.colsurfb.2022.112433

[57] B. Liu , D. Wang , Y. Liu , Q. Zhang , L. Meng , H. Chi , J. Shi , G. Li , J. Li , X. Zhu , Polym. Chem. 2015, 6, 3460.

