# Supplementary material for: A Multi‐Responsive Hydrogel Combined With Mild Heat Stimulation Promotes Diabetic Wound Healing by Regulating Inflammatory and Enhancing Angiogenesis
Source: Adv Sci (Weinh). 2024 Oct 22;11(46):2408783. doi: 10.1002/advs.202408783 (PMC11633493; doi:10.1002/advs.202408783)
Supplement: Supplementary file 1 — Supporting Information [file ADVS-11-2408783-s001.docx]

Supporting Information

A Multi-Responsive Hydrogel Combined with Mild Heat Stimulation Promotes Diabetic Wound Healing by Regulating Inflammatory and Enhancing Angiogenesis

*Fanjia Dai, Jiaying Zhang, Fengjiao Chen, Xianwu Chen, Celine Jessica Lee, Hongze Liang, Lingling Zhao*, Hui Tan**

1. **Fig. S1**
2. **The calculation of the photothermal conversion efficiency**


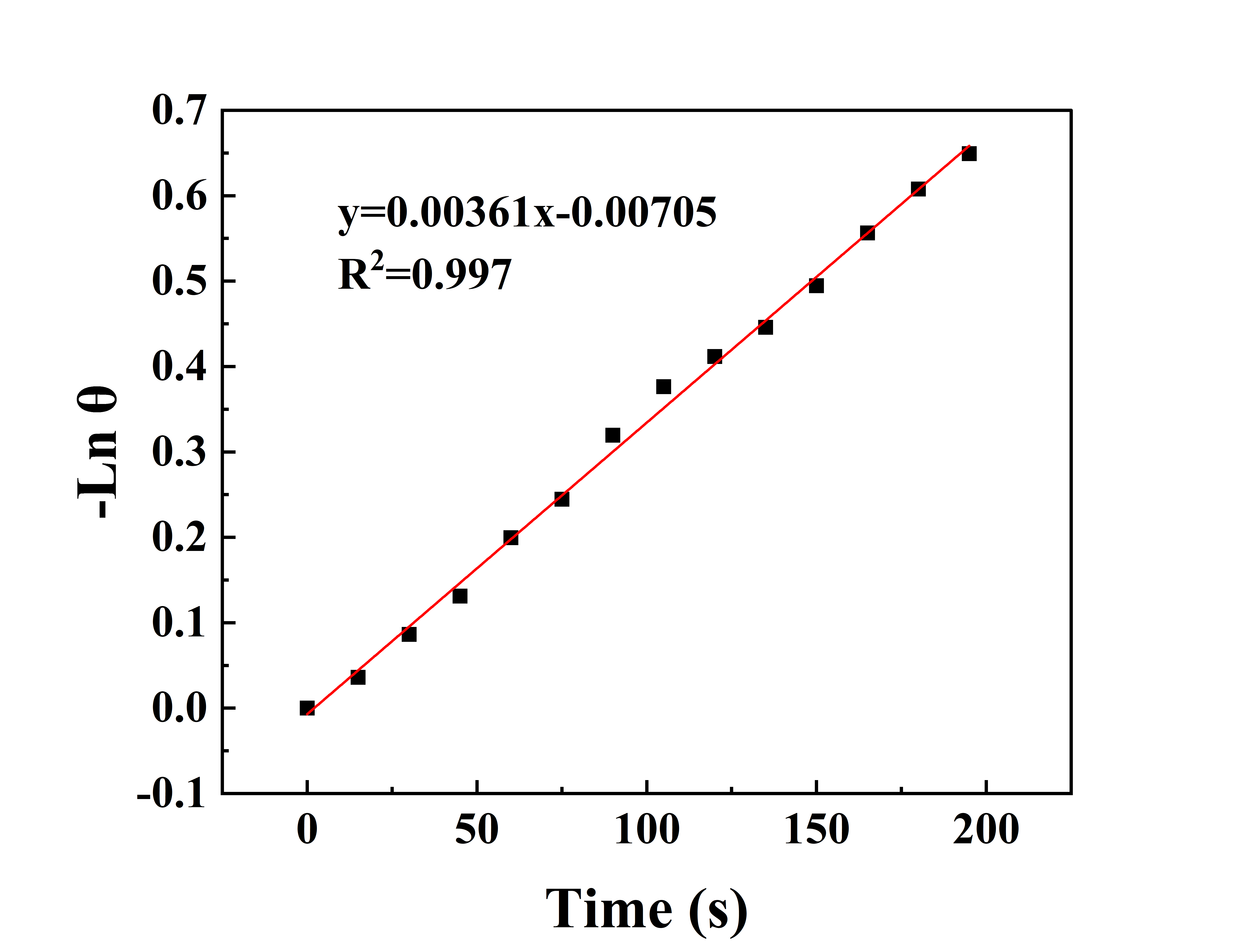


**Fig. S1**. **Liner cooling time data versus negative natural logarithm of driving force temperature (*–ln θ*) with *τs* (CPO/D@P) = 277.01.**

The photothermal conversion efficiency (***η***) is calculated as follow equations (1):

$=\frac{hS\left( T_{max}-T_{surr} \right)-Q_{0}}{I(1-{10}^{-A_{\lambda}}）}$ (1)

*T_max_*: the steady state maximum temperature of the CPO/D@P, *T_surr_*: the ambient room temperature, *I*: the laser power, $A_{\lambda}$: the absorbance of PDA at 808 nm.

*T_max_*=41.4 ℃, *T_surr_*=24.0 ℃, *I*=1 W cm^-2^, $A_{\lambda}$=1.635.

*hS* is obtained from equation [(2)](https://www.sciencedirect.com/science/article/pii/S138589472032310X#e0010), *Q_0_* was calculated from equation (3).

$s=\frac{\left( m_{d}c_{d} \right)}{hS}$ (2)

*τs*: the characteristic thermal time constant, *m_d_*: the weight of the CPO/D@P, *c_d_*: in the best capacity which was about 4.2 J g^−1^ k^−1^, *h*: the heat transfer coefficient, *S*: the sample container surface area.

*m_d_*=1.05 g, *c_d_*=4.2 J·g^-1^·k^-1^.

$Q_{0}=hS\left( T_{max, water}-T_{surr} \right)$ (3)

*Q_0_*: the background energy input without the CPO/D@P, *T_max, water_*: the steady state maximum temperature of water.

*T_max, water_*=27.8 ℃.

Ultimately, *η*=22.17%
